# Health equity impact of community-initiated kangaroo mother care: a randomized controlled trial

**DOI:** 10.1186/s12939-021-01605-0

**Published:** 2021-12-24

**Authors:** Tarun Shankar Choudhary, Sarmila Mazumder, Øystein Ariansen Haaland, Sunita Taneja, Rajiv Bahl, Jose Martines, Maharaj Kishan Bhan, Kjell Arne Johansson, Halvor Sommerfelt, Nita Bhandari, Ole F Norheim

**Affiliations:** 1 Society for Applied Studies, Centre for Health Research and Development, New Delhi, India; 2 Department of Global Public Health and Primary Care, University of Bergen, Bergen, Norway; 3 Department of Maternal, Newborn, Child and Adolescent Health, World Health Organization, Geneva, Switzerland; 4 Centre for Intervention Science in Maternal and Child Health, Department of Global Public Health and Primary Care, University of Bergen, Bergen, Norway; 5 Indian Institute of Technology, New Delhi, India; 6 Bergen Centre for Ethics and Priority Setting, Department of Global Public Health and Primary Care, University of Bergen, Bergen, Norway; 7 Cluster for Global Health, Division for Health Services, Norwegian Institute of Public Health, Oslo, Norway

**Keywords:** Kangaroo Mother Care, Community, Neonatal, Infant, Equity, Randomized controlled trial

## Abstract

**Background:**

Kangaroo mother care (KMC) can substantially enhance overall survival of low birthweight babies. In a large randomized controlled trial, we recently showed that supporting mothers to provide community initiated KMC (ciKMC) can reduce mortality among infants up to 180 days of life by 25% (hazard ratio (HR) 0.75). With the current analysis, we aimed to explore if ciKMC promotion leads to increased inequity in survival.

**Methods:**

In the trial we randomized 8402 low birthweight babies to a ciKMC (4480 babies) and a control (3922 babies) arm, between 2015 and 2018 in Haryana, India. We estimated the difference in concentration indices, which measure inequality, between babies in the ciKMC and control arms for survival until 180 days of life. Further, we compared the effect of ciKMC promotion across subgroups defined by socioeconomic status, caste, maternal literacy, infant’s sex, and religion.

**Results:**

Our intervention did not increase survival inequity, as the concentration index in the ciKMC arm of the trial was 0.05 (95% CI -0.07 to 0.17) lower than in the control arm. Survival impact was higher among those belonging to the lower two wealth quintiles, those born to illiterate mothers and those belonging to religions other than Hindu.

**Conclusions:**

We found that ciKMC promotion did not increase inequity in survival associated with wealth. The beneficial impact of ciKMC tended to be larger among vulnerable groups. Supporting mothers to provide KMC at home to low birthweight babies will not increase and could indeed reduce inequities in infant survival.

**Trial registration:**

ClinicalTrials.gov, NCT02653534. Registered January 12, 2016—Retrospectively registered.

## Background

Health inequality refers to differences in the distribution of health status across population groups. When these differences are preventable and unjust across socially relevant groupings, it leads to health inequity [1]. Reducing inequities in health is a widely endorsed policy goal; however, the empirical evidence base for how to achieve such a reduction is weak [2]. Interest is now shifting from only describing social determinants of health to understanding how these inequities can be addressed using programmatic and policy interventions [3]. Randomized controlled trials (RCTs) typically report average efficacy, and there is a lack of high-quality RCTs describing the impact of interventions on inequities between individuals or groups. Rigorous methods for measuring equity impact on health using an RCT design are needed, as data from RCTs are used to inform policy decisions [3–5].

It is important to investigate whether efficacious interventions lead to inequities. Such information is useful for those deciding on the efficient and equitable allocation of healthcare resources. It could also provide input to economic evaluations, such as distributional cost-effectiveness analysis (DCEA) [6]. There are at least three different ways in which an intervention can be shown to have equity impact in RCTs [4]. First, if an effective intervention targets the most disadvantaged subgroup, improvement in health outcomes will have a positive impact on health equity. Second, if the intervention aims to improve access to health services of known effectiveness, improved access for disadvantaged groups over groups that are better off will also increase equity. Finally, if the health impact is differential across groups defined by equity-relevant characteristics like wealth, literacy, caste, race and gender, there may be a positive impact on equity if disadvantaged groups benefit more than those better off. On the contrary, if an intervention is of greater benefit to advantaged groups than to disadvantaged groups, intervention-generated inequalities may arise.

In general, lower socioeconomic status, poverty, low levels of education and residence in rural or remote areas are all associated with poor health [7]. In India, girls overall, experience poorer health than boys and people belonging to socially disadvantaged castes experience poorer health than others [8, 9]. Low birthweight infants (birthweight < 2,500 g), especially in resource-poor settings, have a much higher mortality and morbidity than infants with a normal birthweight [10]. In a recent RCT in Haryana, India, we found that active promotion of community-initiated kangaroo mother care (ciKMC) for infants weighing 1500 to 2250 g within 72 h of being born reduced their mortality until 180 days of life by 25% (95% CI: 7% to 40%) [11].

Here, we define health inequity as an unequal and unfair distribution of health benefits across strata defined by socioeconomic status, caste, maternal literacy, religion or infant’s sex, whereas a positive equity impact is reflected in a reduction in unequal and unfair occurrence of health outcomes in infants who receive the intervention compared to those who do not [3, 12]. In this study, we explored the impact of ciKMC on the distribution of improvements in survival across wealth status, maternal literacy, caste, religion, and infant sex. We hypothesized that ciKMC would not lead to unfair intervention-generated inequities by disproportionately benefitting advantaged groups.

## Methods

Adhering to the CONSORT-Equity 2017 reporting standards, which aims to improve the reporting of intervention effects in randomized trials where health equity is relevant, we collected our data in Faridabad and Palwal district of Haryana in India between 30 July 2015 and 31 October 2018 as part of the individually randomized controlled parallel-arm ciKMC trial [3, 11, 13]. Earlier studies done in this area show substantial health inequity across characteristics like caste, gender, maternal literacy, and wealth status [9, 13, 14]. Our formative research facilitated capturing the characteristics of the PROGRESS-Plus framework, including caste, gender, religion, education, and socioeconomic status [15, 16]. The PROGRESS-Plus framework is an acronym used to identify characteristics that stratify health opportunities and outcomes [16].

The main effect estimate of the current study was the difference in concentration index between infants in the ciKMC and control arms for post enrolment survival until 180 days of life. We also compared the post-enrolment mortality rate ratios until 180 days of life between the infants in the ciKMC and the control arms across wealth status, maternal literacy, family’s caste, family’s religion, and infant’s sex.

The field team assessed the infants at home and weighed them as soon as possible (no later than 72 h) after birth; they were eligible if they weighed between 1500 and 2250 g [11]. Infants with an inability to feed, difficulty in breathing, less than normal movements or with gross congenital malformation; those for whom KMC had been initiated in hospitals; and infants whose mothers planned to move out of the study area during the trial period were excluded.

The intervention consisted of the newborns being kept in skin-to-skin contact with their mother or a surrogate and exclusively breastfed for as long as possible. An intervention delivery team made nine home visits in the intervention arm during the first 28 days of life to support KMC. No intervention was given to the control families but families in both the intervention and control arms of the trial were expected to receive routine home-based care from the public health system, which comprises of 6 home visits on day 1, 3, 7, 14, 21 and 28 of life [17]. We collected socioeconomic and demographic data at baseline. During regular home visits, a separate team of well-trained research assistants, masked to trial-arm allocation, collected data on mortality of the participating babies until they were six months of age. The data collection procedures were identical in both arms.

### Descriptive statistics with summary measures of health inequality

We used an asset index score, a composite measure of the living standards of the households, to rank the study participants. We calculated the asset index, using data on household ownership of selected assets (e.g., televisions and bicycles), the materials used for housing construction, sanitation facilities and the source of drinking water. Each household asset was assigned a weight or factor score generated through principal components analysis. The resulting asset scores were standardized to a standard normal distribution with a mean of zero and a standard deviation of one. These standardized scores were then used to divide the study population into five quintiles. The method we used to generate the asset index was similar to that used by the Demographic and Health Survey Program [18]. The wealth status of the lower 40% of the study population based on asset index score (i.e., representing the two lowest quintiles) was categorized as poor – the upper three quintiles were categorized as non-poor [19].

We present the study outcomes by wealth quintile to explore social gradients in the two trial arms. To investigate, summarize and draw inferences about the impact of the intervention on health inequity, we used concentration curves, concentration indices and the difference in the concentration indices between the two arms [20]. The concentration curve plots the cumulative proportion of the health variable (y-axis) against the cumulative percentage of the population, ranked by living standards, beginning with the poorest, and ending with the richest (x-axis) [21]. The concentration index is defined as twice the area between the concentration curve and the line of equality (the 45-degree line). So, when there is no socioeconomic-related inequality, the concentration index is zero [20, 21]. We used an F-test to estimate the statistical precision i.e., 95% confidence interval of this difference in concentration index (Δ ci) for mortality up to 180 days of life between the intervention and control arm. A positive Δ ci reflects a positive equity impact i.e., reduced inequity and negative Δ ci indicates increased inequity [22]. The magnitude of the Δ ci is a measure of the extent to which inequity was increased or decreased due to the intervention. We used Stata 16.1 (StataCorp LLC, College Station, Texas) and community-contributed packages (“DASP” and “Lorenz”) for our analyses [23, 24].

### Inferential analysis

We frequency-aggregated the data for death, follow-up time and infants enrolled from the same household across the subgroups defined by wealth status [non-poor vs poor], family caste [scheduled caste (SC)/scheduled tribe (ST)/other backward caste (OBC) vs other], mother’s literacy [illiterate vs literate], infant’s sex, and religion [Hindu vs other]. In each stratum, we then estimated the incidence rate ratios (IRRs) for post-enrolment death during the first half of infancy between the ciKMC and the control arm using log-binomial generalized linear models with follow-up time in child months. We estimated the biologic interaction (i.e., interaction assessed on the additive scale) using the absolute excess rate due to interaction (AErI) for wealth status, infant’s sex, caste, religion, and mother’s literacy status [25, 26] using the appropriate interaction terms in the above-mentioned regression models. The regression analyses accounted for clustering of deaths among infants within the same household using robust standard errors.

## Results

Randomization successfully balanced important PROGRESS-Plus characteristics between the trial arms (Table 1).

**Table 1:** Baseline characteristics of the participants in the ciKMC trial across relevant PROGRESS Plus characteristics

Characteristic	ciKMC (n = 4480) number (%)	Control (n = 3922) number (%)
**Family wealth status**^**a**^		
Non-poor	2714 (61)	2323 (59)
Poor	1761 (39)	1599 (41)
**Family caste**		
Scheduled caste/tribe (SC/ST)	1573 (35)	1305 (33)
Other backward caste (OBC)	1947 (44)	1777 (45)
Other	955 (21)	839 (21)
**Infant’s sex**		
Male	1907 (43)	1741 (44)
Female	2573 (57)	2181 (56)
**Mother literacy**		
Never been to school^a^	1625 (36)	1341 (34)
**Religion**^**a**^		
Hindu	3653 (82)	3195 (82)
Other	827 (18)	727 (18)
Mean (SD) Age of mother years^a^	23.3 (3.7)	23.4 (3.8)
Mother working outside of home^a^	226 (5.0)	223 (5.7)

^a^Data for different characteristics were missing for < 30 (0.4%) of the participants; *SD* Standard deviation.

**Fig. 1:**
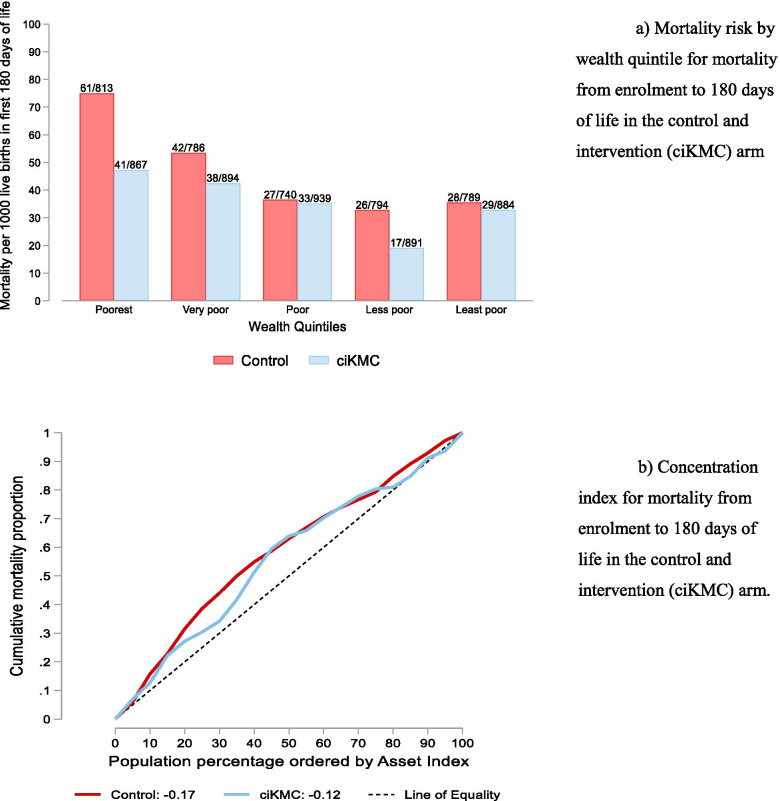
**a** Mortality risk by wealth quintile for mortality from enrolment to 180 days of life in the control and intervention (ciKMC) armQuery. **b** Concentration index for mortality from enrolment to 180 days of life in the control and intervention (ciKMC) arm

There was a clear social gradient with higher mortality in the first six months of life in the poorest compared to the least poor quintile in the control arm (Fig. 1a). From the observed distribution (red curve in Fig. 1b), we can see that 50% of the mortality was concentrated among the poorest third of the study participants. In general, mortality was concentrated among the poorer families, reflected in the distribution curve lying above the diagonal line of equality (black dashed straight curve). The observed concentration index of -0.17 shows that mortality was concentrated among the poorer participants.

We assessed the equity impact of the intervention on mortality by comparing the distributions in the control arm with the corresponding distributions in the intervention arm (Fig. 1a and b). Mortality from enrolment to 180 days of life was lower in the intervention arm compared to in the control arm for all quintiles, that difference being most pronounced for the poorest quintile (Fig. 1a). The mortality in the intervention arm (solid blue curve in Fig. 1b was still concentrated among the poorest families, but to a lesser degree than in the control arm. The concentration index in the intervention arm was -0.12, i.e., 0.05 (95% CIs -0.07 to 0.17) lower compared to the control arm. Hence, ciKMC did not increase inequity in early infant survival (Table 2).

**Table 2:** Concentration Index (95% CI) for mortality from enrolment to 180 days of life and its difference, Δ ci (95% CI) between the intervention and control arm

Outcome	Control Concentration Index Estimate (95% CI)	ciKMC Concentration Index Estimate (95% CI)	Δ ci (95% CI)
Mortality from enrolment to 180 days of life	-0.17 (-0.25 to -0.09)	-0.12 (-0.21 to -0.03)	0.05(-0.07 to 0.17)

Δ ci = difference in concentration index between the intervention and control arm

### Impact of the intervention across subgroups

Table 3 contains the comparisons between the ciKMC and the control arm IRRs for death from enrolment to 180 days of life across socioeconomic status (poor vs non-poor), caste (SC/ST/OBC vs other), maternal literacy (illiterate vs literate), sex (boy vs girl) and religion (Hindu vs other). ciKMC promotion substantially reduced mortality in all the disadvantaged subgroups. The IRRs tended to be lower, i.e., the survival benefit of ciKMC promotion higher, among babies of illiterate (vs. those of literate) mothers and among babies born into poorer (vs. into non-poor) families. Thus, infants in the intervention arm born in poor families had a lower IRR than those in non-poor families (0.70 vs 0.85). Likewise, mortality saw a greater decrease with the intervention in disadvantaged castes versus in other castes (IRR: 0.73 vs 0.95), among infants born to illiterate vs literate mothers (IRR: 0.65 vs 0.86), among female vs male infants (IRR: 0.74 vs 0.80) and among other religions vs Hindus (IRR: 0.69 vs 0.79). Still, the 95% CIs for the AErIs were relatively wide. The positive – albeit statistically not very precise – interaction between the PROGRESS Plus subgroups and allocation to the ciKMC trial arm suggests that the effect of the impact of ciKMC may be greater in disadvantaged groups (Table 3).

**Table 3:** Incidence Rate Ratio (95% CI) and Absolute excess rate due to interaction (AErI) (95% CI) for death between enrolment to day 180 of life in the intervention and control arm of the ciKMC trial^a^ across religion, caste, maternal literacy, infant’s sex, and socioeconomic status

Mortality from enrolment to 180 days					
		Control Deaths (child months under observation)	ciKMC Deaths (child months under observation)	IRR (95% CI)	AErI (95% CI) (child-months under observation)
**Socio-economic status **^**b**^					
Poor		102 (8499)	79 (9372)	0.70 (0.56, 0.88)	0.0026 (0.0001, 0.0053)
Non-poor		81 (12,267)	79 (14,061)	0.85 (0.66, 1.09)	
**Caste**					
SC ^c^/ST ^c^/OBC ^d^		154 (16,234)	127 (18,359)	0.73 (0.53, 1.00)	0.0023 (-0.0016, 0.0061)
Other		29 (4533)	31 (5074)	0.95 (0.63, 1.45)	
**Maternal literacy**					
Illiterate		89 (7020)	71 (8575)	0.65 (0.52, 0.81)	0.0034 (0.0006, 0.0063)
Literate		94 (13,746)	87 (14,858)	0.86 (0.65, 1.12)	
**Infant’s sex**					
Female	103 (11,540)		89 (13,527)	0.74 (0.49, 1.10)	0.0006 (-0.0039, 0.0052)
Male	80 (9226)		69 (9907)	0.80 (0.55, 1.17)	
**Religion**					
Other	45 (3775)		35 (4271)	0.69 (0.49, 0.96)	0.0020 (-0.0021, 0.0062)
Hindu	138 (16,992)		123 (19,162)	0.79 (0.58, 1.08)	

^a^Data for different characteristics were missing for < 30 (0.4%) of the participants.

^b^Lower two wealth quintiles have been categorised as poor and the top three wealth quintiles have been categorised as non-poor.

^c^Scheduled castes (*SCs*), and scheduled tribes (*STs*) are officially designated groups of historically disadvantaged people in India.

^d^Other backward caste (*OBC*) is a collective term the Government of India uses to classify castes that are educationally or socially disadvantaged.

## Discussion

We found that ciKMC provided to low birthweight infants (1500 to 2250 g) did not seem to increase inequity in survival across categories of wealth, socioeconomic status, caste, maternal literacy, infant’s sex, or religious group. If anything, promotion of ciKMC reduced inequity, as illustrated by the reduction in the concentration index and the fact that the effect of ciKMC on survival appeared to be greater among infants from poor households, infants born to illiterate mothers and infants from disadvantaged castes. Still, the CIs were generally too wide to conclude that ciKMC promotion reduces inequity.

To our knowledge, no other large equity relevant RCTs have been conducted on KMC. This study is the first and the largest RCT assessing the mortality impact of KMC initiated in communities where low birthweight is very common. Less than 0.03% of the participating infants were lost to follow-up. The only other RCT on ciKMC that has been conducted did not explore the consistency of impact across potential axes of socio-economic differences [27]. The Cochrane Review on the effect of hospital initiated KMC was updated in 2016; however, subgroup analyses and other analyses to examine possible negative equity impact were not presented [28].

A limited number of studies have directly estimated the equity impact of interventions using individual-level data from randomized trials. We applied the classical measures of health equity and added a direct measure of inequity impact using the difference in concentration index along with a regression-based analysis of biologic interaction to assess the equity impact [20, 25]. The indices illustrate the distribution of intervention health impact.

While it is comforting that promotion of ciKMC did not seem to induce inequity in infant survival, there are several reasons to expect that ciKMC in fact can reduce it. For example, a reduction in inequity is consistent with findings that providing access to healthcare close to home reduces health-related inequities [29]. Lack of education among mothers has been associated with reduced awareness regarding the importance of exclusive breastfeeding [30]. We believe that the very close physical contact between baby and mother could supersede any tendencies of suboptimal breastfeeding practices which may be more common among women with less education. Further, KMC has been credited with empowering mothers and increasing their self-efficacy to take better care of their infants [27]. Finally, as in other parts of the world, the proportion of children born with low birthweight is higher in disadvantaged groups in the study area [9, 31]. Many parents in these groups have limited awareness of appropriate newborn care and have poor access to facility-based neonatal care, which is often unaffordable for them. Hence, ciKMC could have a higher impact on survival in these groups than in advantaged groups.

We used an asset index to rank individuals to assess the inequality in health outcomes; and the caution that applies to such assessment should be exercised in interpreting our findings [32, 33]. Despite the large sample size (n = 8402), the trial was not primarily powered to assess the differential impact of the intervention across axes of disadvantage. Still, the large trial enabled us to state that ciKMC promotion and support did not enhance pre-existing inequities in young infant survival. Low birthweight was common in the trial setting, and caution must be exercised in extrapolating the findings to settings with high neonatal mortality but a lower prevalence of low birthweight.

Limited statistical precision will be an important issue for future studies on equity impact of healthcare interventions targeting important but rare outcomes like death. In addition to an intervention being offered to everyone, there may be a need to target those known to be particularly vulnerable to undesirable outcomes and/or who are especially resistant to adequate uptake or responding to an intervention. Although outcomes with higher incidence/prevalence would be more suitable for such studies, equity impact analyses for rare outcomes of clear importance such as those addressed in the current study are valuable – they would convey whether potential intervention-driven inequities are generated or not.

## Conclusion

In addition to substantially reducing mortality, promoting ciKMC did not induce a discernible inequity in survival-related to wealth, caste, maternal literacy, infant sex or religion between enrolment and 180 days of life. The intervention placed little demand on family resources beyond the mother’s time; it did not require any equipment or complex knowledge of technical skills; and it was culturally appropriate – these are all characteristics that facilitated the intervention adoption by poorer and less-educated families. Supporting mothers to provide KMC at home to low birthweight babies will not increase and could indeed reduce inequities in infant survival.

## Data Availability

The dataset pertaining to the results reported in the manuscript will be made available to others only for health and medical research, subject to constraints of the consent under which the data was collected. De-identified individual participant data will be made available along with the data dictionary, study protocol, and informed consent form. Data will be available beginning 12 months and ending 5 years after publication of this article. Requests for data should be made to Dr Tarun Shankar Choudhary (tarun.choudhary@sas.org.in). The requester should provide a methodologically sound secondary research proposal, approved by an independent review committee. The requester must be able to show their ability to carry out the proposed use of the requested dataset through their peer review publications and declare conflicts of interest in relation to the requested dataset and their funding sources. The authors reserve the right to refuse sharing of data in the face of potential adversarial conflicts of interest. A Data Sharing Agreement that meets the data sharing requirements of the Society for Applied Studies (New Delhi, India) and Centre for International Health, University of Bergen (Norway) will be signed with the data requester. Data must only be used for the purpose described in the secondary research proposal as further stipulated in the Data Sharing Agreement. Data will be transferred only to requesters named in the original proposal and as specified in the relevant Data Sharing Agreement.

## Abbreviations

RCTs: Randomized controlled trials
DCEA: Distributional Cost-Effectiveness Analysis
ciKMC: Community Initiated Kangaroo Mother Care
CONSORT: Consolidated Standards of Reporting Trials
PCA: Principal Component Analysis
HR: Hazard Ratio
SC: Scheduled Caste
ST: Scheduled Tribe
OBC: Other Backward Caste
AErI: Absolute Excess rate due to Interaction
CI: Confidence Interval
